# 3-*O*-Methyldopa inhibits astrocyte-mediated dopaminergic neuroprotective effects of l-DOPA

**DOI:** 10.1186/s12868-016-0289-0

**Published:** 2016-07-25

**Authors:** Masato Asanuma, Ikuko Miyazaki

**Affiliations:** 1 Department of Medical Neurobiology, Okayama University Graduate School of Medicine, Dentistry and Pharmaceutical Sciences, 2-5-1 Shikata-cho, Kita-ku, Okayama, 700-8558 Japan; 2 Department of Brain Science, Okayama University Graduate School of Medicine, Dentistry and Pharmaceutical Sciences, Okayama, 700-8558 Japan

**Keywords:** Astrocyte, l-DOPA, 3-*O*-Methyldopa, Glutathione, Entacapone

## Abstract

**Background:**

We evaluated the effects of 3-*O*-methyldopa (3-OMD), a metabolite of l-DOPA which is formed by catechol-*O*-methyltransferase (COMT), on the uptake, metabolism, and neuroprotective effects of l-DOPA in striatal astrocytes. We examined changes in the numbers of dopaminergic neurons after treatment with l-DOPA and 3-OMD or entacapone, a peripheral COMT inhibitor, using primary cultured mesencephalic neurons and striatal astrocytes.

**Results:**

The number of tyrosine hydroxylase-positive dopaminergic neurons was not affected by l-DOPA treatment in mesencephalic neurons alone. However, the increase in viability of dopaminergic neurons in the presence of astrocytes was further enhanced after methyl-l-DOPA treatment (25 µM) in mixed cultured mesencephalic neurons and striatal astrocytes. The neuroprotective effect of 25 µM l-DOPA was almost completely inhibited by simultaneous treatment with 3-OMD (10 or 100 µM), and was enhanced by concomitant treatment with entacapone (0.3 µM). The uptake of l-DOPA into and the release of glutathione from striatal astrocytes after l-DOPA treatment (100 µM) were inhibited by simultaneous exposure to 3-OMD (100 µM).

**Conclusions:**

These data suggest that l-DOPA exerts its neuroprotective effect on dopaminergic neurons via astrocytes and that 3-OMD competes with l-DOPA by acting on target molecule(s) (possibly including glutathione) released from astrocytes. Since some amount of entacapone can cross the blood–brain barrier, this reagent may enhance l-DOPA transportation by inhibiting COMT and increase the astrocyte-mediated neuroprotective effects of l-DOPA on dopaminergic neurons.

**Electronic supplementary material:**

The online version of this article (doi:10.1186/s12868-016-0289-0) contains supplementary material, which is available to authorized users.

## Background

Astrocytes are abundant neuron-supporting glial cells that harbor a powerful arsenal of neuroprotective antioxidants and neurotrophic factors [1, 2]. However, astrocytes have not as yet been used as targets for clinical therapeutic drug development for the treatment of Parkinson’s disease (PD). We have previously revealed that some reagents targeting astrocytes have neuroprotective effects against dopaminergic neurodegeneration in Parkinson’s mouse models. These compounds enhanced astrocyte proliferation and upregulated the production of antioxidant molecules, such as glutathione (GSH) and metallothionein, in astrocytes [3, 4]. Furthermore, our recent study indicated that striatal astrocytes act as a reservoir for l-DOPA, which is the most widely used drug in patients with PD. Specifically, we observed that astrocytes uptake or release l-DOPA depending on extracellular l-DOPA concentrations without converting it to dopamine (DA) [5]. Although it is still unresolved whether l-DOPA is toxic to accelerate degeneration of DA neurons in PD patients, it is the most therapeutically efficacious drug for the treatment of PD to date [6]. l-DOPA has been shown to increase GSH levels, exert neurotrophic effects, and protect neurons from oxidative stress in mesencephalic neuronal and glial mixed cultures, but not in neurons alone [7, 8].

3-*O*-Methyldopa (3-OMD), a major metabolite of l-DOPA, is formed by catechol-*O*-methyltransferase (COMT) in peripheral tissues and in the brain [9]. It accumulates in the plasma and the brain because of its longer half-life (15 h) than l-DOPA (1 h) [10]. 3-OMD competitively inhibits l-DOPA transport into the brain at the blood–brain barrier [11, 12]. It also inhibits l-DOPA accumulation in the striatum by inhibiting aromatic amino acid decarboxylase activity, and not by inhibiting the firing of dopaminergic neurons [13]. Furthermore, 3-OMD inhibits DA release from rat striatal slices [14]. Thus, 3-OMD competitively inhibits the pharmacodynamics of l-DOPA and dopamine.

In order to clarify whether 3-OMD affects astrocyte-mediated effects of l-DOPA, we evaluated the uptake and metabolism of l-DOPA in striatal astrocytes, the release of GSH and neurotrophic factors from astrocytes, and the neuroprotective effects of l-DOPA mediated by striatal astrocytes. We used primary cultured mesencephalic neurons and striatal astrocytes treated with methyl-l-DOPA, a water-soluble form of l-DOPA which can be rapidly converted to l-DOPA in culture medium [5], and 3-OMD. We also examined the effects of entacapone, a peripheral COMT inhibitor, on l-DOPA-induced changes in the numbers of dopaminergic neurons in a mixed culture of neurons and astrocytes.

## Methods

### Materials

Methyl-l-DOPA ester hydrochloride (methyl-l-DOPA, D1507) and 3-OMD monohydrate (3-methoxy-l-tyrosine, M4255) were purchased from Sigma-Aldrich (St. Louis, MO). Entacapone was obtained from Orion Pharma (Espoo, Finland).

### Animals

All animal procedures described in our experiments were in strict accordance with the Guidelines for Animal Experiments of Okayama University Advanced Science Research Center, and were approved by the Animal Care and Use Committee of Okayama University Advanced Science Research Center. Special care was taken to minimize the number of animals used. For primary striatal or mesencephalic cell cultures, pregnant Sprague–Dawley rats were purchased from Charles River Japan, Inc. (Yokohama, Japan).

### Cell culture

Primary striatal and mesencephalic cell cultures were prepared as described previously [15]. Briefly, we dissected striatal and mesencephalic tissue from Sprague–Dawley rat embryos at 15 days of gestation. After trypsin treatment, cells from the striatum or the mesencephalon were incubated with 0.004 % deoxyribonuclease I and 0.03 % trypsin inhibitor (Sigma-Aldrich) at 37 °C for 7 min. After centrifugation (420*g* × 5 min), the cell pellet was gently resuspended in 4 ml of Dulbecco’s Modified Eagle Medium (Gibco BRL, Rockville, MD) supplemented with 10 % fetal bovine serum, 4 mM l-glutamine, and 60 µg/ml kanamycin sulfate. The dissociated cells were plated onto six-well culture plates or four-chamber glass culture slides coated with poly-d-lysine (Becton–Dickinson Labware, Bedford, MA) at a density of 2 × 10^5^ cells/cm^2^. For mesencephalic neuron-enriched cultures (mesencephalic neurons), we replaced the medium with fresh medium supplemented with 2 µM cytosine-β-d-arabinofuranoside (Sigma-Aldrich) to inhibit the replication of non-neuronal cells 24 h after initial plating of the dissociated cells. We then incubated the cells for five more days. Preliminary studies confirmed that 95 % of the neuron-rich cultures were immunoreactive for the neuronal marker microtubule-associated protein 2. For striatal glial-enriched cell cultures (striatal astrocytes), dissociated cells were incubated in the same culture medium for 7 days, then subcultured onto six-well culture plates (3.6 × 10^4^ cells/cm^2^) and incubated for another 7, 2 and 7 days for the measurement of GSH, glial cell line derived neurotrophic factor (GDNF), and basic fibroblast growth factor (bFGF), respectively. Alternatively, we subcultured the astrocytes onto 100-mm dishes (6.4 × 10^4^ cells/cm^2^) for the measurement of l-DOPA and DA, and incubated them for another 14 days. Over 95 % of the cultured glial-enriched cells showed immunoreactivity to glial fibrillary acidic protein. For neuron-astrocyte cocultures, striatal astrocytes were seeded at a density of 2 × 10^4^ cells/cm^2^ directly onto a layer of mesencephalic neurons that were already cultured in 4-chamber slides for 4 days, as described. The cell mixture was then cocultured for another 2 days. All cultures were maintained at 37 °C in a 5–95 % CO_2_–air gas mixture.

### Treatments

Mesencephalic neurons or a mixed culture of mesencephalic neurons and striatal astrocytes on four-chamber culture slides were treated with methyl-l-DOPA (25 µM) and/or 3-OMD monohydrate (10 or 100 µM), or with methyl-l-DOPA (25 µM) and/or entacapone (0.03, 0.1, or 0.3 µM) for 24 h prior to immunohistochemistry for the detection of dopaminergic neurons. Striatal astrocytes grown on 6-well plates were treated with methyl-l-DOPA (25 or 100 µM) and/or 3-OMD (10 or 100 µM) for 24 h and the concentrations of GSH in astrocytes and GSH released from astrocytes were measured. For the measurement of GDNF and bFGF, astrocytes were cultured on 6-well plates for 2 days for GDNF measurement or 7 days for bFGF measurement, and then treated with methyl-l-DOPA (25 µM for GDNF, 50 µM for bFGF) and/or 3-OMD (10 or 100 µM) in serum-free medium for 24 h. To measure the concentrations of l-DOPA, and DA and its metabolites in astrocytes, striatal astrocytes were treated with methyl-l-DOPA (100 µM) and/or 3-OMD (10 µM) for 4 h. To measure the levels of GSH, GDNF, and bFGF released from astrocytes, we collected glia conditioned medium (GCM) after a 24-h treatment with methyl-l-DOPA and/or 3-OMD, centrifuged it at 3000×*g* for 3 min to remove cellular debris, and stored the supernatant at −80 °C until use.

### Immunohistochemistry

Cells on chamber slides were fixed with 4 % paraformaldehyde (Nakarai, Kyoto, Japan) for 20 min, washed with 10 mM phosphate-buffered saline (PBS, pH 7.4), and blocked with 2.5 % normal goat serum (Vector Laboratories, Burlingame, CA) for 20 min at 23 °C. Cells were then incubated with rabbit anti-tyrosine hydroxylase (TH) antibody (1:1000 dilution, Protos Biotech Corporation, New York, NY) diluted in 10 mM PBS containing 0.1 % Triton X-100 (0.1 % PBST) for 18 h at 4 °C. The secondary antibody was goat anti-rabbit IgG conjugated to Alexa Fluor 488 (1:200 dilution, Molecular Probes, Eugene, OR). The cells were counterstained with Hoechst nuclear stain (10 µg/ml, Molecular Probes) for 2 min and washed before mounting with Fluoromounting medium (Dako Cytomation, Glostrup, Denmark).

All slides were observed under a fluorescence microscope (Olympus BX50-FLA, Tokyo, Japan) using a mercury lamp using a 470–490 nm or a 360–370 nm band-pass filter to excite Alexa Fluor 488 or Hoechst dye, respectively. Light emitted from Alexa Fluor 488 or Hoechst dye was collected through a 515–550 nm band-pass filter or a 420 nm long-pass filter, respectively. Adobe Photoshop CS4 software (Adobe, Waltham, MA) was used for digital amplification of the images.

### Determination of total GSH

GSH levels in cultured cells and GCM were determined by the enzymatic recycling method of Tietze [16] with some modifications [3]. To prepare the samples, homogenized cells in 0.1 M phosphate buffer (pH 7.4) or GCM were treated with equivalent volumes of 10 % trichloroacetic acid. After centrifugation, the acid extracts were mixed with 0.01 M phosphate buffer (pH 7.4, 174 µl), NADPH (4 mM, 15 µl), GSH reductase (6 U/ml, 30 µl, Wako Pure Chemical, Tokyo, Japan) and 5,5′-dithiobis-2-nitrobenzoic acid (10 mM, 15 µl, Wako), and incubated at 37 °C. The formation of 2-nitro-5-thiobenzoic acid was measured by absorbance at 412 nm. Total GSH was determined using a standard curve that was constructed using known amounts of GSH.

### Measurements of GDNF and bFGF

The levels of GDNF and bFGF released from astrocytes in serum-free GCM were measured by enzyme-linked immunosorbent assay (ELISA) using a GDNF Emax Immunoassay System (Promega, Madison, WI, USA) and an ELISA kit for Rat Fibroblast Growth Factor 2, Basic (FGF2) (Uscn Life Science Inc., Wuhan, China) according to the manufacturer’s protocols. Aprotinin (10 µg/ml) and leupeptin (1 µg/ml) were added to serum-free GCM collected from methyl-l-DOPA and/or 3-OMD-treated striatal astrocytes.

The GDNF in GCM was captured by monoclonal anti-GDNF antibody precoated onto a 96-well plate and colorimetrically detected using a polyclonal anti-GDNF antibody, horseradish peroxidase (HRP)-conjugated anti-IgY, and the chromogenic substrate 3,3′,5,5′-tetramethylbenzidine (TMB). The concentration of GDNF was quantified by measuring the absorbance at 450 nm and comparing to a GDNF standard. bFGF in GCM and biotin-labeled bFGF were competitively captured by the monoclonal anti-bFGF antibody precoated on a well and the bound biotinylated bFGF was detected via its subsequent reaction with avidin-conjugated HRP and the chromogen TMB. As before, the absorbance was measured at 450 nm. The concentration of bFGF in GCM was determined using a reverse proportional standard curve.

### Measurement of intracellular l-DOPA, and DA and its metabolites

The contents of l-DOPA, and DA and its metabolites, 3-OMD, 3,4-dihydroxyphenyl acetic acid (DOPAC) and homovanillic acid (HVA), were measured in astrocytes treated with methyl-l-DOPA and/or 3-OMD using high-performance liquid chromatography with an electrochemical detector (HPLC-ECD), as described previously [5]. Striatal astrocytes treated with methyl-l-DOPA (100 µM) and/or 3-OMD (10 µM) for 4 h were homogenized using 5 volumes of 200 mM ice-cold perchloric acid containing 10 mM ethylenediaminetetraacetic acid (EDTA, Dojindo, Kumamoto, Japan). After centrifugation (11,750×*g*, 20 min at 4 °C), the supernatant was filtered (0.45 µm) and then injected directly into an HPLC-ECD (Tosoh Co., Tokyo). The HPLC system consisted of a delivery pump (PX-8020, Tosoh Co.) and an analytical column (EICOMPAK SC-5ODS, 3.0 mm × 150 mm, Eicom Co., Kyoto, Japan). An electrochemical detector (EC-8020, Tosoh Co.) with glassy carbon at a voltage setting of 700 mV and an Ag/AgCl reference electrode were used. A mobile phase containing 0.1 M citrate-sodium acetate buffer (pH 3.5), methanol (17 % v/v), EDTA-2Na, and sodium 1-octanesulfonate was infused at a flow rate of 0.6 ml/min. Regarding the detection limit, the peak less than 3 mV/min was not detected. Extraction efficiency was approximate 25 % by measuring internal control isoproterenol which was put into cell preparation before homogenization.

### Statistical analysis

Results are presented as mean ± standard error of the mean (SEM). Between-group differences were analyzed by one-way analysis of variance (ANOVA), followed by post hoc Fisher’s PLSD test. A *p* value less than 0.05 indicated the presence of a statistically significant difference.

## Results

### Striatal astrocyte-mediated effects of l-DOPA and 3-OMD on mesencephalic dopaminergic neurons

**Fig. 1:**
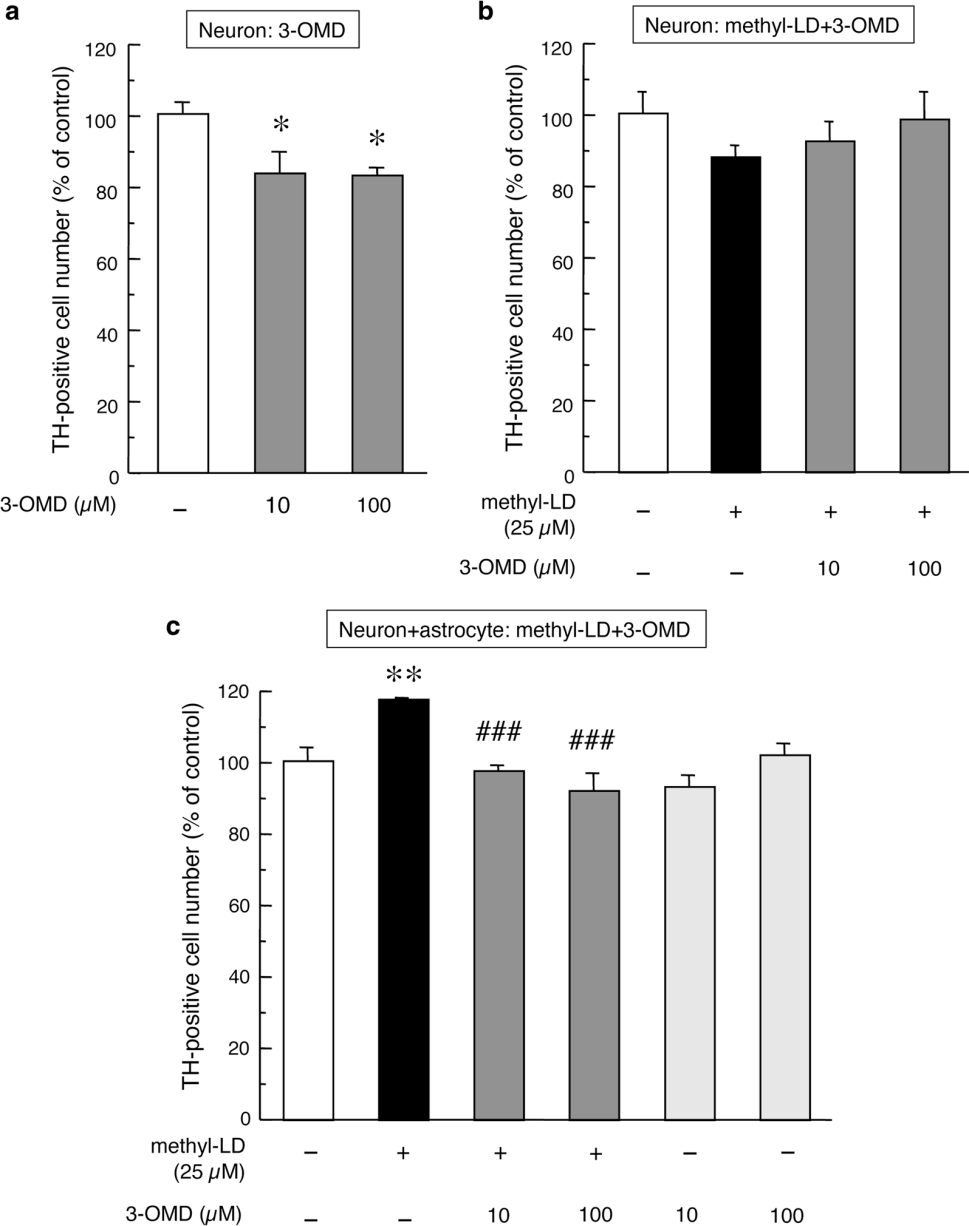
Changes in the number of TH-positive cells in primary cultured mesencephalic neurons (**a**, **b**) or in mixed cultured mesencephalic neurons with striatal astrocytes (**c**) after the treatment with 3-OMD (10, 100 µM) alone (**a**) or methyl-l-DOPA (25 µM) and 3-OMD (10, 100 µM) (**b**, **c**) for 24 h. Each value is mean ± SEM of total number of TH-positive neurons from different 4 chambers (n = 4), which is expressed as percentage of control. *p < 0.05, **p < 0.01 versus vehicle-treated control, ^###^p < 0.001 versus methyl-l-DOPA-treated group

Primary cultured mesencephalic neurons alone are vulnerable, with gradually decreasing cell numbers during cultivation. Treatment with 3-OMD (10 or 100 µM) enhanced this vulnerability and significantly decreased the numbers of mesencephalic DA neurons (Fig. 1a). In mesencephalic neurons alone, methyl-l-DOPA (25 µM), with or without 3-OMD (10 or 100 µM) showed no effects on the number of TH-positive DA neurons (Fig. 1b). The viability of DA neurons is increased by the presence of astrocytes [17]. In mesencephalic neurons co-cultured with striatal astrocytes, methyl-l-DOPA (25 µM) further increased the viability of mesencephalic TH-positive dopaminergic neurons (Fig. 1c). Neither methyl-l-DOPA nor 3-OMD affected total number of neurons (data not shown). The neuroprotective effect of methyl-l-DOPA (25 µM) in mixed cultures was almost completely inhibited by concomitant treatment with 3-OMD (10 or 100 µM) for 24 h (Fig. 1c). We recently revealed that striatal astrocytes act as a reservoir of l-DOPA and govern the uptake or release of l-DOPA depending on extracellular l-DOPA concentrations [5]. Therefore, we examined the effects of 3-OMD on l-DOPA uptake and metabolism into/in striatal astrocytes. We observed marked increases in the concentration of l-DOPA and 3-OMD in striatal astrocytes after a 4-h methyl-l-DOPA (100 µM) treatment (Table 1). In the present study, l-DOPA was not converted to DA and its metabolites DOPAC and HVA in astrocytes as previously reported [5]. Furthermore, we performed measurement of l-DOPA, DA and its metabolites using other HPLC-ECD system (EICOM 300, Eicom Co., Kyoto). However, we again failed to detect DA and its metabolites DOPAC and HVA in astrocytes (data not shown). The failure of detecting DA and its metabolites indicates that aromatic amino acid decarboxylase (AADC) appears inactive in the astrocytes [5]. The exposure to 3-OMD (10 µM) increased its level in the astrocytes. Simultaneous exposure to 10 µM 3-OMD significantly inhibited the uptake of l-DOPA into astrocytes, but showed just additive effects on the level of 3-OMD in the astrocytes (Table 1).

**Table 1:** Concentration of l-DOPA, dopamine and their metabolites in striatal astrocytes 4 h after methyl-l-DOPA (100 µM) and/or 3-OMD (10 µM) exposure

	Control	3-OMD	Methyl-l-DOPA	3-OMD + methyl-l-DOPA
l-DOPA	N.D.	N.D.	411.0 ± 12.1**	347.0 ± 15.6**^,^^##^
DA	N.D.	N.D.	N.D.	N.D.
DOPAC	N.D.	N.D.	N.D.	N.D.
HVA	N.D.	N.D.	N.D.	N.D.
3-OMD	N.D.	12.5 ± 1.02**	15.3 ± 1.57**	26.0 ± 0.83**^,^^##^^,^^$$^

Striatal astrocytes were treated with methyl-l-DOPA (100 µM) and/or 3-OMD (10 µM) for 4 h. The concentrations of l-DOPA, DA, DOPAC, HVA and 3-OMD were measured by HPLC assay. N.D: not detected. Data (pmol/mg protein) are presented as mean ± SEM (n = 4)

* p < 0.05, ** p < 0.001 versus control group

^##^ p < 0.001 versus methyl-l-DOPA-treated group

^$$^p < 0.001 versus 3-OMD-treated group

### Effects of l-DOPA and 3-OMD on GSH or its release in/from striatal astrocytes

**Fig. 2:**
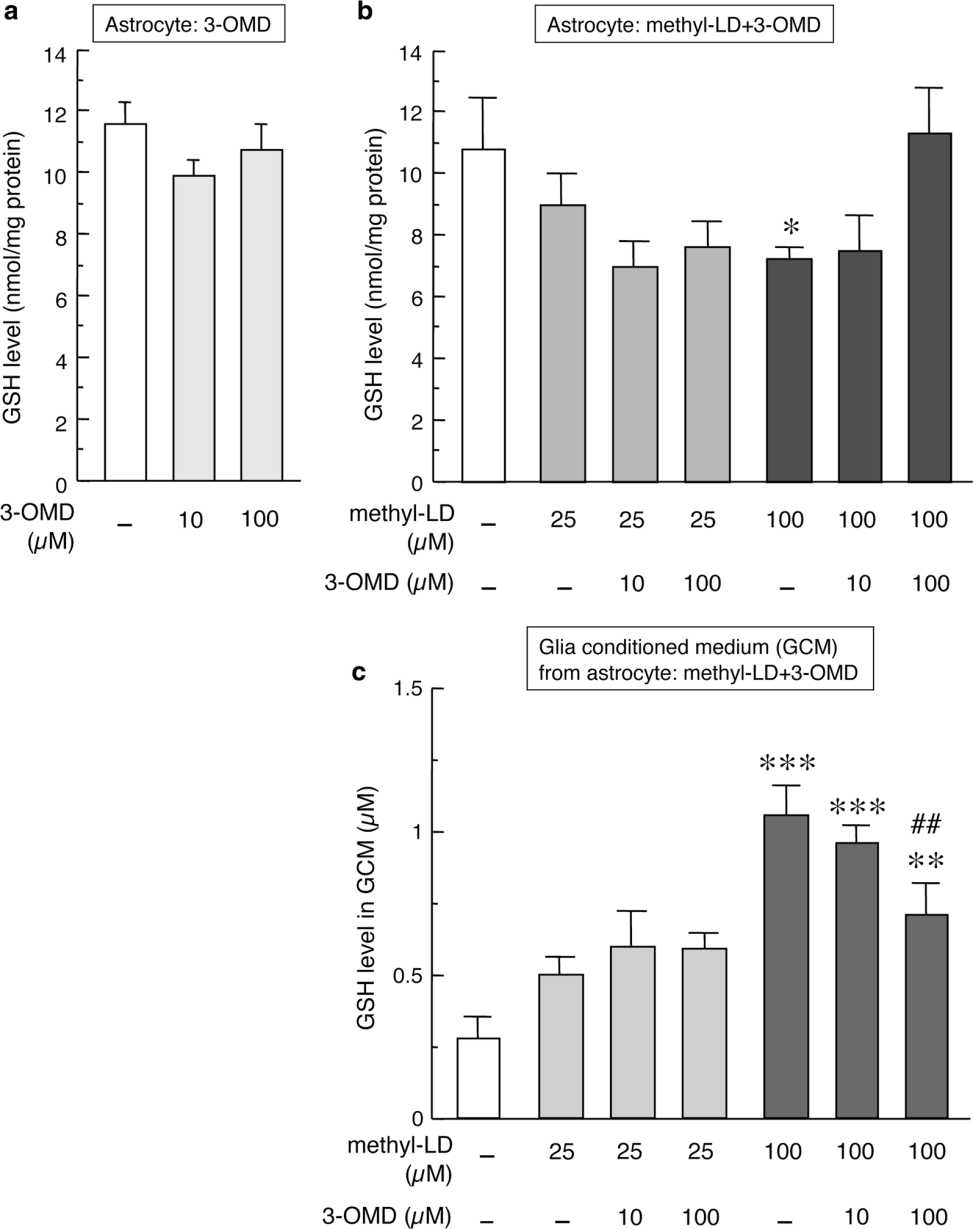
GSH levels in striatal astrocytes (**a**, **b**) or in their GCM (**c**) after the treatment with 3-OMD (10 or 100 µM) alone (**a**) or methyl-l-DOPA (25 or 100 µM) and 3-OMD (10 or 100 µM) (**b**, **c**) for 24 h. Data are mean ± SEM (n = 6). *p < 0.05, **p < 0.01, ***p < 0.001 versus control vehicle-treated, ^##^p < 0.01 versus methyl-l-DOPA-treated group

The neuroprotective effects of l-DOPA on mesencephalic DA neurons are dependent on the presence of striatal astrocytes. It has been reported that l-DOPA increases GSH levels to protect neurons against oxidative stress in neuron-astrocyte mixed cultures [7, 8], and that GSH synthesis in neurons is dependent on GSH synthesis in astrocytes and its release [18, 19]. To investigate possible factors involved in the astrocyte-mediated neuroprotective effects of l-DOPA, we examined changes in the levels of GSH in astrocytes and its release from astrocytes after methyl-l-DOPA and/or 3-OMD treatment. Exposure to 3-OMD had no effects on GSH levels in striatal astrocytes (Fig. 2a). Treatment with 100 µM methyl-l-DOPA for 24 h, and to a lesser extent at the lower dose 25 µM, significantly decreased GSH contents in astrocytes (Fig. 2b) and increased GSH levels in the GCM, indicating the release of GSH from astrocytes (Fig. 2c). This l-DOPA (100 µM)-induced GSH release from striatal astrocytes was counteracted by concomitant treatment with 100 µM 3-OMD (Fig. 2b, c). We also measured GDNF and bFGF released from astrocytes into the GCM following treatment with methyl-l-DOPA and/or 3-OMD. Treatment with 25 µM methyl-l-DOPA for 24 h increased the levels of GDNF in the GCM. This increase was not affected by simultaneous exposure to 3-OMD (Additional file 1: Fig. S1A). Neither lower (25 µM: data not shown) and higher (50 µM) doses of methyl-l-DOPA nor 3-OMD affected the levels of bFGF in the GCM (Additional file 1: Fig. S1B).

### Effects of entacapone on l-DOPA-induced striatal astrocyte-mediated neuroprotection

**Fig. 3:**
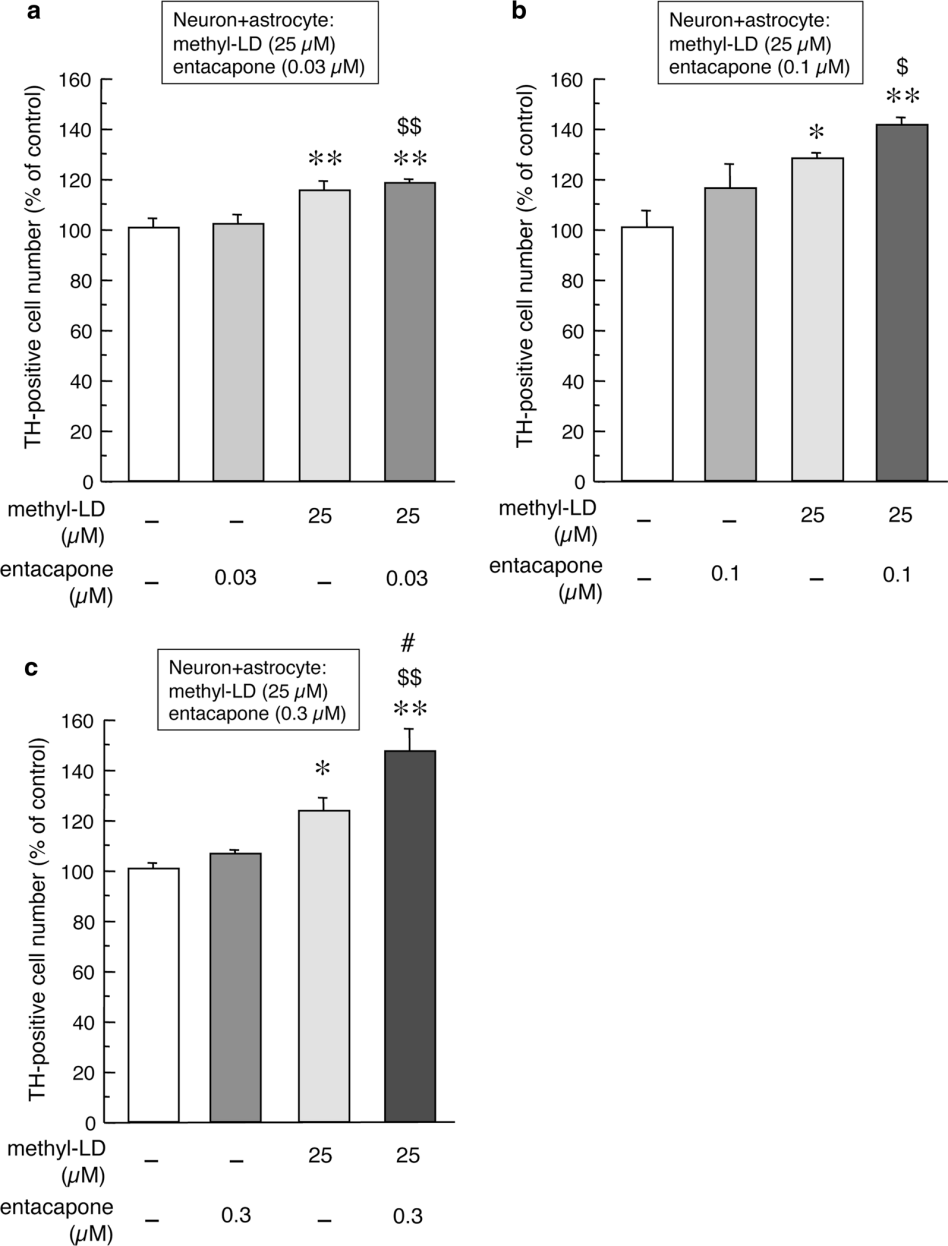
Effects of entacapone on methyl-l-DOPA (25 µM)-induced changes in the number of TH-positive cells in mixed primary cultured mesencephalic neurons with striatal astrocytes. Mixed neurons and astrocytes were treated with methyl-l-DOPA (25 µM) and entacapone (0.03, 0.1 or 0.3 µM: **a**, **b** or **c**, respectively) for 24 h. Values are mean of the number of TH-positive cells ± SEM (n = 4 chambers) expressed as percentage of control. *p < 0.05, **p < 0.01 versus vehicle-treated control, ^#^p < 0.05 versus methyl-l-DOPA-treated group, ^$^p < 0.05, ^$$^p < 0.01 versus entacapone-treated group

The conversion of l-DOPA to 3-OMD is inhibited by the peripheral COMT inhibitor entacapone. Since small amounts of entacapone can cross the blood–brain barrier, we examined whether entacapone can enhance the astrocyte-mediated neuroprotective effects of l-DOPA in the present study. As well as the results in Fig. 1c, the treatment with 25 µM methyl-l-DOPA for 24 h increased the number of TH-positive dopaminergic neurons in mixed cultures of mesencephalic neurons and striatal astrocytes (Fig. 3a–c). Concomitant treatment with entacapone enhanced l-DOPA-induced increases in the numbers of dopaminergic neurons in a dose-dependent manner, especially when used at 0.3 µM (Fig. 3c). The single entacapone treatment showed no effects on the number of TH-positive neurons in primary cultured mesencephalic neurons alone in the absence of astrocytes (data not shown).

## Discussion

In the present study, we examined the effects of the l-DOPA metabolite 3-OMD, which is formed by COMT, and the peripheral COMT inhibitor entacapone on the astrocytic uptake, metabolism, and neuroprotective effects of l-DOPA. The main findings of this study are: (1) methyl-l-DOPA treatment (25 µM) increases the viability of DA neurons in mixed cultures of mesencephalic neurons and striatal astrocytes, but not in mesencephalic neurons alone, (2) the neuroprotective effects of l-DOPA on mesencephalic DA neurons are inhibited by simultaneous treatment with 3-OMD, (3) increased l-DOPA uptake into astrocytes and the GSH release from striatal astrocytes after methyl-l-DOPA treatment are significantly inhibited by simultaneous exposure to 3-OMD, and (4) the neuroprotective effects of l-DOPA in the presence of astrocytes are enhanced by concomitant treatment with entacapone.

Mesencephalic neurons, especially TH-positive DA neurons, are vulnerable to oxidative stress. The viability of primary cultured DA neurons gradually decreases when they are cultivated in the absence of astrocytes. However, the vulnerability of DA neurons is ameliorated by the presence of astrocytes [17]. In the present study, l-DOPA treatment (25 µM) increased the viability of DA neurons co-cultured with striatal astrocytes, suggesting that the neuroprotective effects of l-DOPA are exerted via astrocytes. We recently demonstrated that striatal astrocytes act as regulators of extracellular l-DOPA concentrations by their uptake or release of l-DOPA [5]. It is well known that 3-OMD competitively inhibits the pharmacodynamics of l-DOPA at various points, including its transport at the blood–brain barrier and its uptake into and release from neurons [11, 12, 14]. Also, in the present study, 3-OMD inhibited l-DOPA uptake into astrocytes, but showed no effect on the intracellular metabolism of l-DOPA in striatal astrocytes. Our results indicate that 3-OMD also counteracted the effects of l-DOPA in astrocytes. Specifically, it inhibited the uptake of l-DOPA into astrocytes and reduced the astrocyte-mediated neuroprotective effects of l-DOPA.

The neurotoxic effects of 3-OMD was not in a dose-dependent manner (Fig. 1a). This implies that DA neurons have an intrinsic protective mechanism against 3-OMD, which is independent of astrocytes. Another possible mechanism is that up-taking of 3-OMD is saturated by low dose of 3-OMD. These may cause small protective effects of presence of astrocytes.

Treatment with proper doses of l-DOPA has been reported to protect neurons against oxidative stress through the up-regulation of GSH production in neuron-astrocyte mixed cultures [7, 8], although excess doses of l-DOPA have been shown to be neurotoxic [2, 20]. In the present study, the optimal dose of l-DOPA significantly increased the viability of DA neurons (Figs. 1, 3), while excess dose of l-DOPA rather declined the number of DA neurons in the neuron-astrocyte mixed cultures (data not shown). It is known that GSH synthesis in neurons is dependent on its synthesis in and release from astrocytes [18, 19]. Previous reports suggest that l-DOPA-induced astrocytic GSH synthesis and release are possible key neuroprotective events. We therefore examined astrocytic GSH contents and the release off GSH from astrocytes following methyl-l-DOPA and/or 3-OMD treatment. l-DOPA treatment promoted GSH release from striatal astrocytes, which was inhibited by simultaneous exposure to 3-OMD. In the control and lower dose l-DOPA-treated groups, total amount of GSH, that is intracellular level (Fig. 2b) plus released extracellular level of GSH (Fig. 2c), is constant. However, higher dose of l-DOPA (100 µM) increased total amount of GSH, suggesting l-DOPA promoted GSH synthesis as well as GSH release. We also measured the levels of the neurotrophic factors GDNF and bFGF released from astrocytes. The release of GDNF from astrocytes was enhanced by l-DOPA treatment, but was not affected by 3-OMD. These data suggest that l-DOPA exerts its neuroprotective effects on DA neurons via astrocytes and that 3-OMD competes with l-DOPA by acting on target molecule(s)—possibly including GSH, but not GDNF or bFGF—released from astrocytes.

The COMT-catalyzed production of 3-OMD from l-DOPA is inhibited by peripheral COMT inhibitors such as entacapone and tolcapone, which hardly cross the blood–brain barrier. However, small amount of peripherally administered entacapone are transported into the brain and distributed into the striatum (approximately 0.2 % of plasma concentration) [21], although the penetration of entacapone into brain tissue is less than that of tolcapone. We therefore expected that the entacapone reaching the brain would inhibit 3-OMD formation and enhance the astrocyte-mediated neuroprotective effects of l-DOPA. As expected, concomitant treatment with low concentrations of entacapone (0.03–0.3 µM) enhanced the l-DOPA (25 µM)-induced increases in the numbers of DA neurons in a dose-dependent manner. This effect was especially prominent at the 0.3 µM dose. This suggests that entacapone may enhance l-DOPA transportation not only by inhibiting 3-OMD formation, but also by altering the astrocyte-mediated neuroprotective effects of l-DOPA on DA neurons.

## Conclusion

Here we report that l-DOPA treatment increases l-DOPA uptake by striatal astrocytes and astrocytic GSH release and exerts its neuroprotective effect on DA neurons in the presence of astrocytes. Furthermore, the astrocyte-mediated effects of l-DOPA were counteracted by concomitant treatment with 3-OMD and enhanced by entacapone treatment. 3-OMD exposure inhibited astrocytic l-DOPA uptake and GSH release. Taken together, the present data suggest that 3-OMD competes with l-DOPA by acting on target molecule(s) (possibly including GSH) released from astrocytes. Since small amounts of entacapone, which promotes l-DOPA transport by inhibiting COMT, can cross the blood–brain barrier, it may enhance the astrocyte-mediated neuroprotective effects of l-DOPA on DA neurons. Further in vivo examination is needed to confirm the neuroprotective effects of l-DOPA and entacapone.

## Additional file

10.1186/s12868-016-0289-0 Levels of GDNF (A) or bFGF (B) in GCM of striatal astrocytes after the treatment with methyl-l-DOPA (25 or 50 µM) and/or 3-OMD (10 or 100 µM) for 24 h. Data are mean ± SEM (n = 6). **p < 0.01 versus control vehicle-treated.

## Abbreviations

ANOVA: analysis of variance
bFGF: basic fibroblast growth factor
COMT: catechol-*O*-methyltransferase
DA: dopamine
DOPAC: 3,4-dihydroxyphenyl acetic acid
ECD: electrochemical detector
EDTA: ethylenediaminetetraacetic acid
ELISA: enzyme-linked immunosorbent assay
GCM: glia conditioned medium
GDNF: glial cell line derived neurotrophic factor
GSH: glutathione
HPLC: high-performance liquid chromatography
HRP: horseradish peroxidase
HVA: homovanillic acid
3-OMD: 3-*O*-methyldopa
PBS: phosphate-buffered saline
PD: Parkinson’s disease
TMB: 3,3′,5,5′-tetramethylbenzidine
TH: tyrosine hydroxylase
