# Supplementary figures and images for: 3-O-Methyldopa inhibits astrocyte-mediated dopaminergic neuroprotective effects of l-DOPA
Source: BMC Neurosci. 2016 Jul 25;17:52. doi: 10.1186/s12868-016-0289-0 (PMC4960704; doi:10.1186/s12868-016-0289-0)

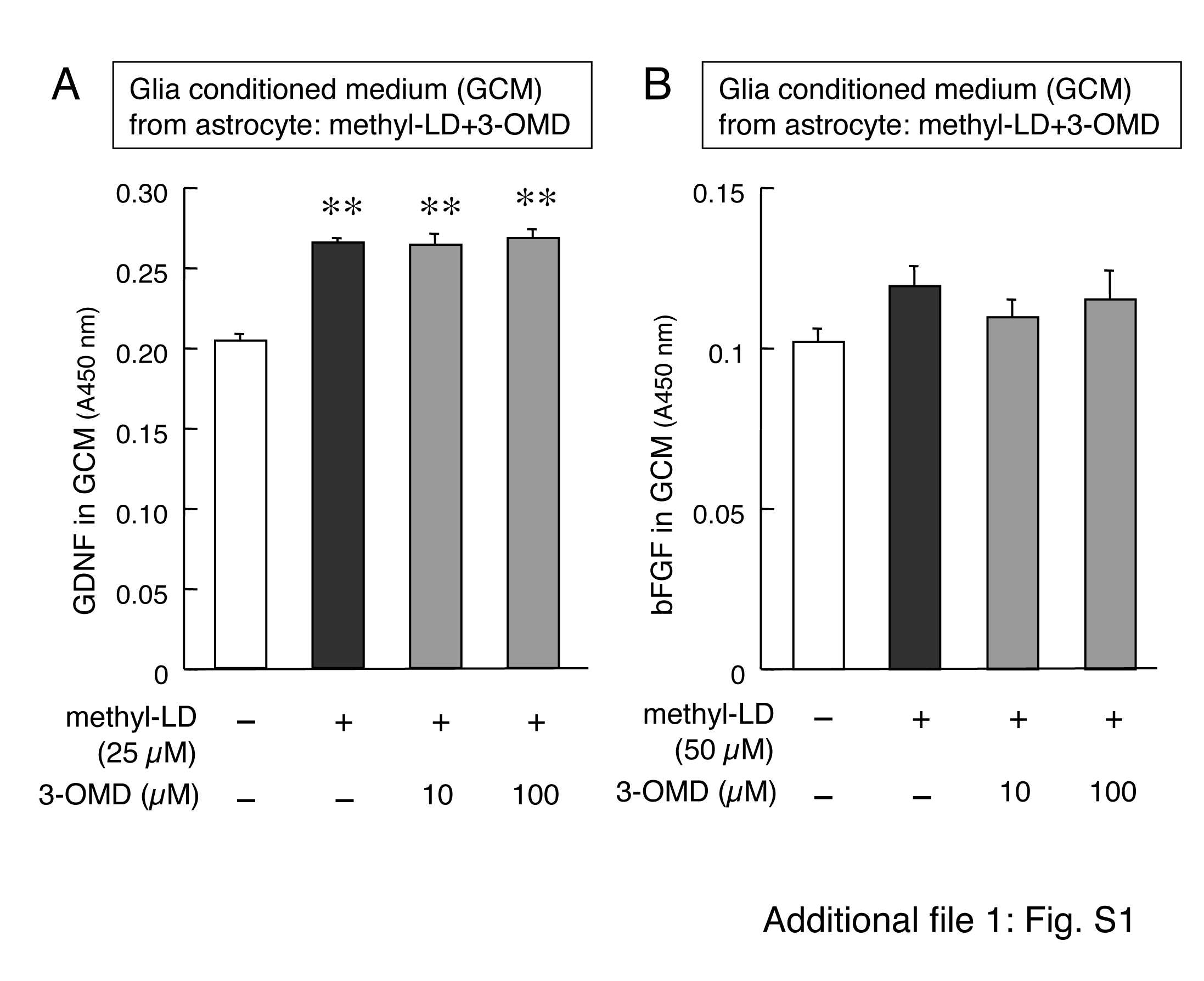

Supplement: Supplementary file 1 — 10.1186/s12868-016-0289-0 Levels of GDNF (A) or bFGF (B) in GCM of striatal astrocytes after the treatment with methyl-l-DOPA (25 or 50 µM) and/or 3-OMD (10 or 100 µM) for 24 h. Data are mean ± SEM (n = 6). **p < 0.01 versus control vehicle-treated. [file 12868_2016_289_MOESM1_ESM.tif]
